# Supplementary material for: Bacteria evoke alarm behaviour in zebrafish
Source: Nat Commun. 2019 Aug 23;10:3831. doi: 10.1038/s41467-019-11608-9 (PMC6707203; doi:10.1038/s41467-019-11608-9)
Supplement: Supplementary file 1 — Supplementary Information [file 41467_2019_11608_MOESM1_ESM.pdf]

Bacteria evoke alarm behaviour in zebrafish

Chia et al.

Supplementary Information

Supplementary Table 1. Bacterial strains tested

Lysate name	Genus/species	Strain ID
Pseu	*Pseudomonas aeruginosa*	ZOR0057
Koc	*Kocuria sp*	ZOR0020
Sta1	*Staphylococcus sp*	ZOR0016
Sta2	*Staphylococcus sp*	ZWU0021
Ent	*Enterococcus gallinarum*	NCDO 2313
Vib1	*Vibrio sp*	ZWU0020
Aer1	*Aeromonas sp*	ZOR0001
Aer2	*Aeromonas sp*	ZOR0039
Aer3	*Aeromonas sp*	ZOR0002
Vib2	*Vibrio sp*	ZOR0035

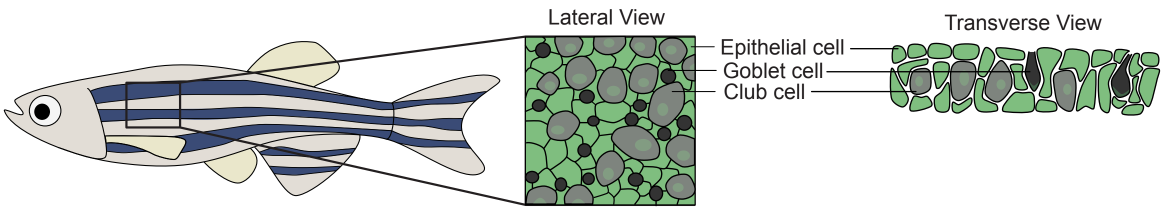

Supplementary Fig. 1. Schematic diagram of cell types in the adult zebrafish skin. Superficial epithelial cells and goblet cells are in contact with the surface, whereas club cells are not.

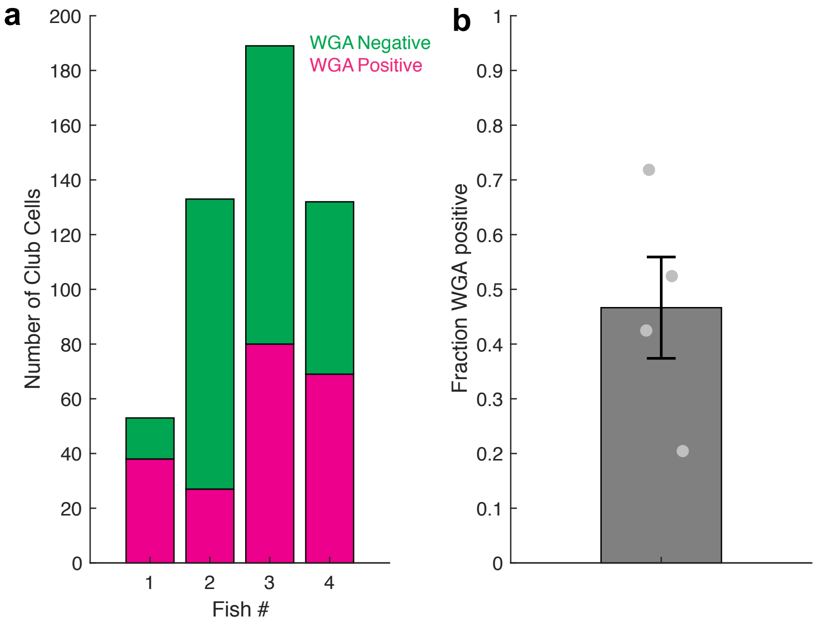

Supplementary Fig. 2 Uptake of mucus by club cells. a Number of club cells with (magenta) and without (green) Alexa594-WGA puncta, following 2 hour incubation in the lectin followed by 2 days in clean water. **b** Mean of the proportion of club cells with WGA puncta. Error bar indicates standard error of the mean.

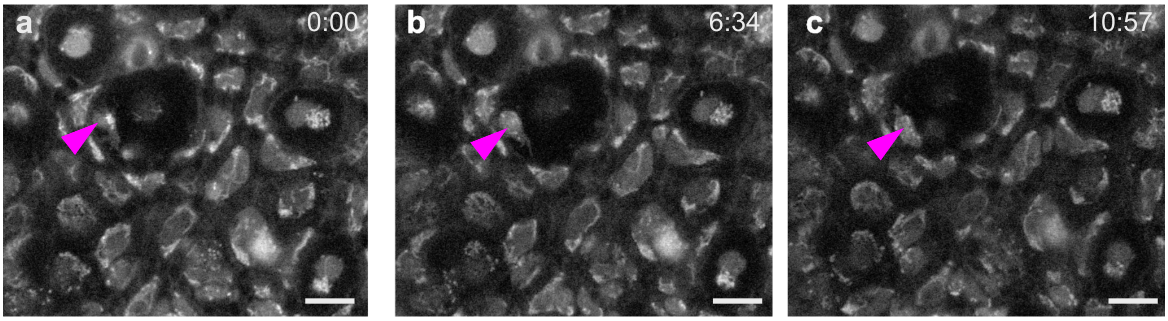

Supplementary Fig. 3. A motile cell within a zebrafish club cell. a-c. Three frames from a time-lapse recording of the skin of fish labelled with SYTO 9. The arrow indicates a cell that changes morphology.

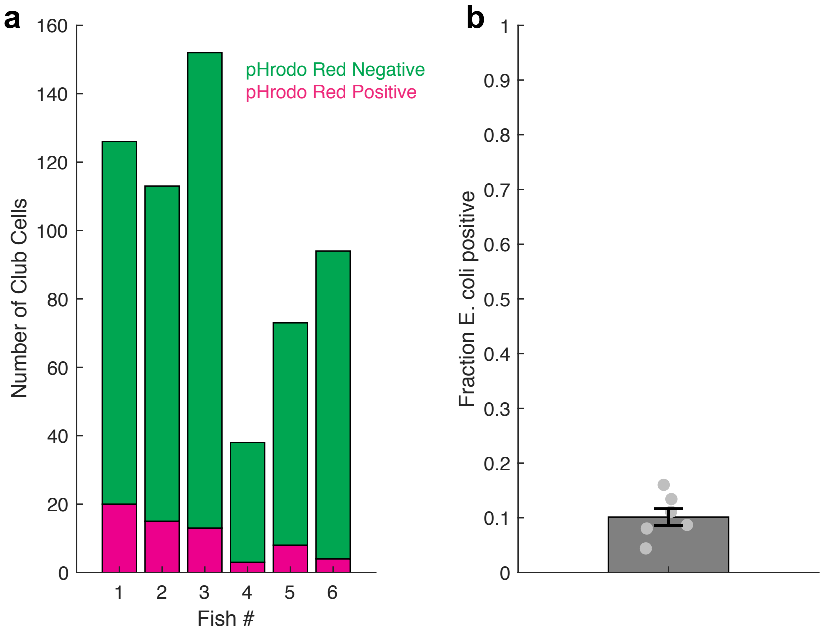

Supplementary Fig. 4. Uptake of *E. coli* by club cells. a. Number of club cells with (magenta) and without (green) diffuse cytoplasm label, following transient incubation in pHrodo Red labelled *E. coli*. **b.** Proportion of club cells with diffuse pHrodo Red fluorescence

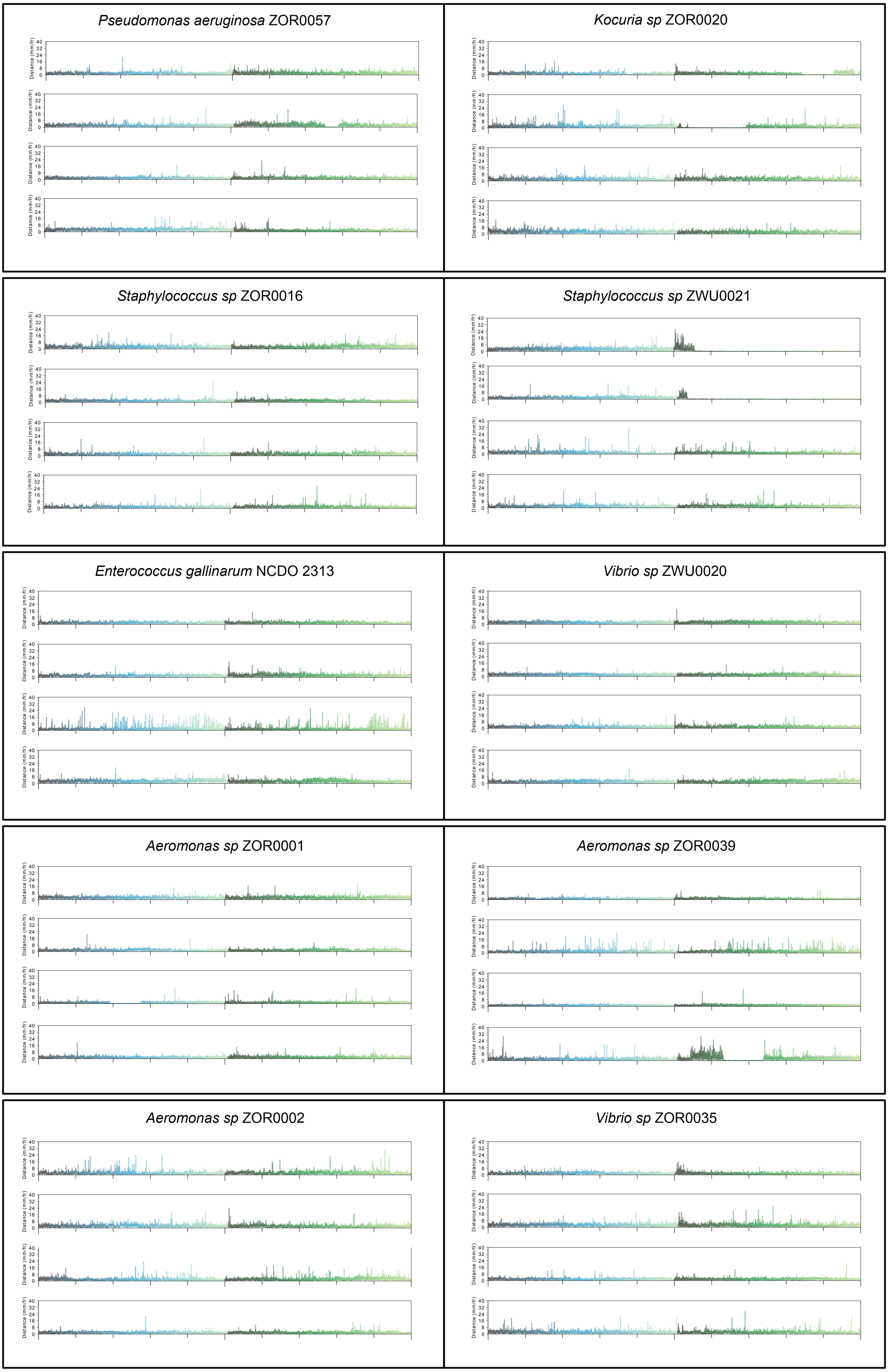

Supplementary Fig. 5. Effect of bacterial lysate on fish speed. Traces showing the speed of 4 fish exposed to each sample. The bacterial lysate was introduced after 5 minutes, and the fish were recorded for a further 5 minutes. Traces are colour-coded according to time.

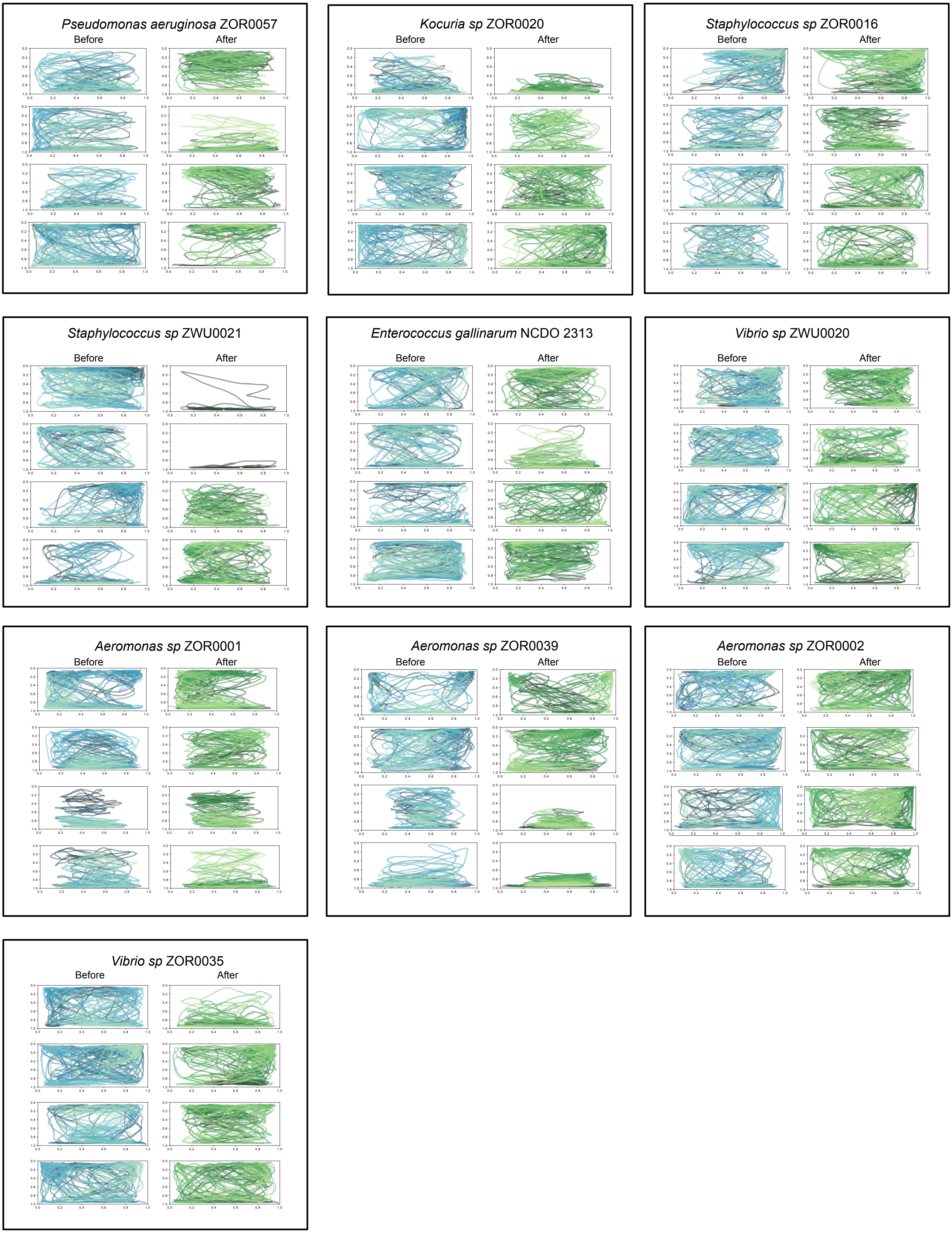

Supplementary Fig. 6. Effect of bacterial lysate on vertical position. Traces showing the position of fish before (left trace) and after (right trace) delivery of bacterial lysate. 4 fish were tested with each lysate. Traces are colour coded according to time, with the same LUT used in Supplementary Fig.5.

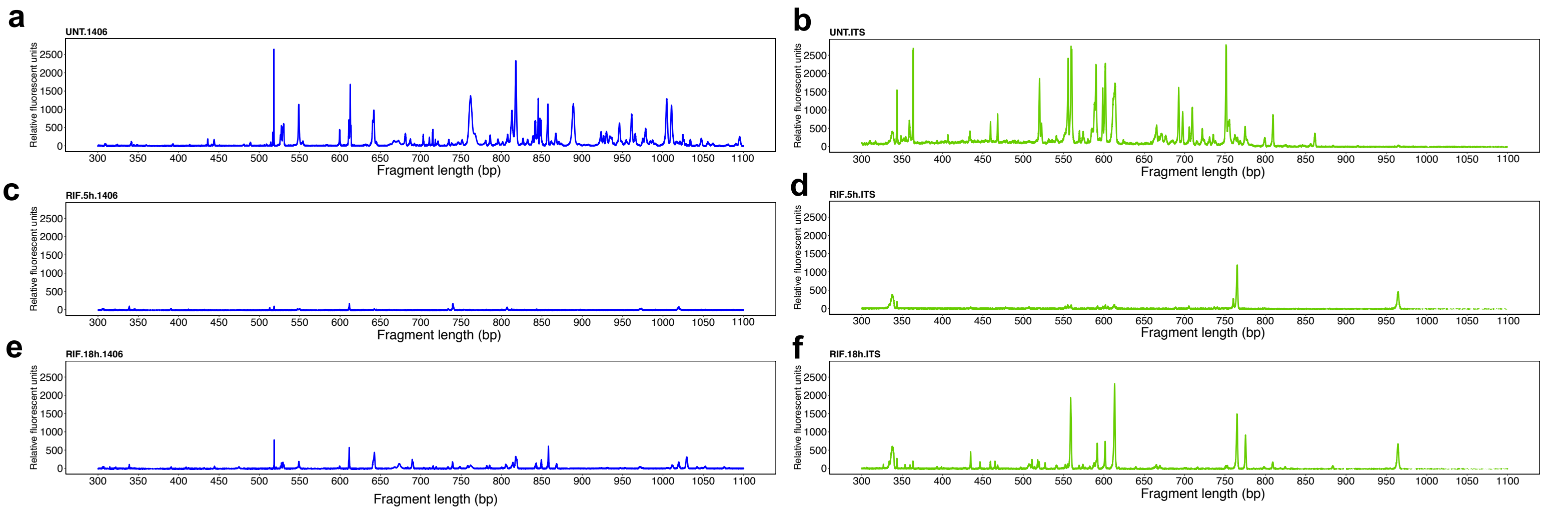

Supplementary Fig. 7. Transient reduction of skin bacteria with antibiotic treatment. a-f. ARISA of swabs from zebrafish skin, with the 1406f/26Sr (a, c, e) and ITSF/ITSReub (b, d, f) primers. **a, b.** Multiple peaks were detected before treatment with the antibiotic rifampicin. **c, d.** 5 hours after treatment, most peaks were absent. **e, f.** 18 hours after the start of treatment, peaks were detected again.

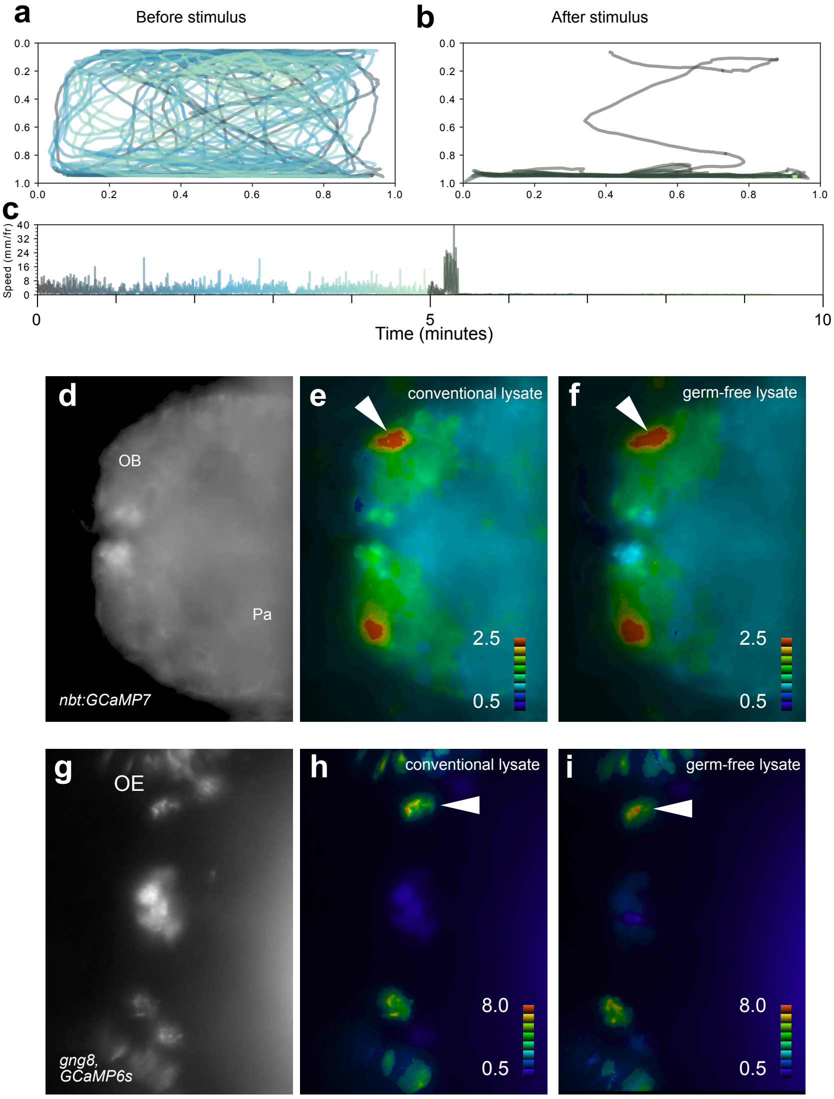

Supplementary Fig. 8 Response to lysate from larval zebrafish. a-c. Response of an adult zebrafish to lysate from 7 dpf larvae. **a, b**. Position of the fish, as viewed from the side, before (a) and after (b) delivery of the larval lysate. **c**. Speed of the fish. There is an increase in speed, followed by freezing, after lysate delivery at the 5-minute time point. **d-f.** The anterior forebrain of a larvae with broad expression of jGCaMP7f. Lysate from conventionally grown (e) and germ-free (f) fish both strongly activate a single glomerulus in the dorso-lateral bulb (arrowhead), as indicated by the high f/f0 ratio in only one region of the bulb. **g-i.** A larvae expressing a calcium indicator under the control of the gng8:GAL4 driver. Lysate from conventionally grown (h) and germ-free (i) larvae both strongly activate OSNs terminating in a single glomerulus in the dorso-lateral bulb (arrowhead). Panels d and g show the average of the time-series; Panels e, f, h and i show the panel with the maximum increase in fluorescence following delivery of the stimulus. All images are dorsal view, with anterior to the left. The wedge indicates the look-up table used, with the numbers indicating the change in fluorescence relative to baseline. OE: olfactory epithelium; OB: olfactory bulb.
