# Supplementary material for: Bacteria evoke alarm behaviour in zebrafish
Source: Nat Commun. 2019 Aug 23;10:3831. doi: 10.1038/s41467-019-11608-9 (PMC6707203; doi:10.1038/s41467-019-11608-9)
Supplement: Supplementary file 3 — Description of Additional Supplementary Files [file 41467_2019_11608_MOESM3_ESM.pdf]

Description of Additional Supplementary Files

File Name: Supplementary Movie 1
Description: **Localization of mucus after transient labelling.** A z-stack of the skin of a fish 2 hours after incubation in Alexa-594 wheat germ agglutinin (magenta). Label is visible on superficial epithelial cells (white arrowheads), and in goblet cells (red arrowheads). Nuclei are labeled by Syto9 (green).

File Name: Supplementary Movie 2
Description: **Localization of mucus 2 days after transient label.** A z-stack of the skin of a fish 2 days after a 2-hour incubation in Alexa-594 WGA. In addition to label in superficial cells and goblet cells, puncta can be seen in club cells (arrows) and also in scattered deep cells. See Fig. 1c.

File Name: Supplementary Movie 3
Description: **Zebrafish club cells can contain a second cell.** Z-stack through the epithelium of a fish labelled with Syto9 (green) and Alexa-568 Phalloidin (magenta). The club cell in the center contains an internalized cell. See Fig. 1m.

File Name: Supplementary Movie 4
Description: **Transfer of Alexa-594 WGA to the cytoplasm of a club cell**. Z-stack showing a club cell with diffuse label of WGA (red arrowhead). At a deeper plane, strong WGA label is visible (yellow arrowhead). See Figs. 1p-x. This fish had been transiently incubated in Alexa-594 WGA, then placed in clean water for 2 days.

File Name: Supplementary Movie 5
Description: **Motile cell within a club cell.** Z-stack through the epithelium of an adult, labelled with SYTO 9. The yellow arrowhead indicates a club cell containing two additional cells, which appear to move. See Supplementary Fig. 3 for several time points at one plane, showing movement of one of these cells.

File Name: Supplementary Movie 6
Description: **Scattered label of club cells in *mpx:GFP* fish.** Z-stack through the epithelium of a fish expressing GFP in neutrophils. Neutrophils, which are motile, are located deep in the epithelium. The cytoplasm of a few club cells are fluorescent at different intensities.

File Name: Supplementary Movie 7
Description: **Trafficking of *E. coli* to club cells.** Z-stack of the skin of a fish that had been transiently placed in water containing fluorescent E. coli (magenta), followed by overnight incubation in clean water. Label is punctate in surface epithelial cells, but diffuse in the cytoplasm of the three club cells indicated by yellow arrowheads. Other club cells (red arrowheads) have no label in the cytoplasm. Cyan arrowheads indicate motile cells. Fish cells are labelled by SYTO 9 (green). See Fig. 3a.

File Name: Supplementary Movie 8
Description: **Uptake of labelled *Staphylococcus aureus*.** Z-stack of the skin of a fish that had been transiently placed in water containing fluorescent *S. aureus* (magenta), followed by

overnight incubation in clean water. The arrows indicate two club cells with internalized bacteria. Fish cells are labelled by SYTO 9 (green). See Fig. 3h, i.

File Name: Supplementary Movie 9
Description: **Endogenous bacteria can be detected in club cells.** Z-stack of a region of the skin, following in situ hybridization with the EUB338 probe. Labelled puncta (arrow) are visible in lower focal planes of the club cell.

File Name: Supplementary Movie 10
Description: **Time-lapse recording of the skin of an adult *actb2:LIFEACT-GFPe114* fish.** The reporter is expressed primarily in motile cells of the adult skin. Smaller cells resemble neutrophils, while larger cells resemble macrophages. Time indicated in min:sec.
