# Supplementary material for: Bacteria evoke alarm behaviour in zebrafish
Source: Nat Commun. 2019 Aug 23;10:3831. doi: 10.1038/s41467-019-11608-9 (PMC6707203; doi:10.1038/s41467-019-11608-9)
Supplement: Supplementary file 14 — Reporting Summary [file 41467_2019_11608_MOESM14_ESM.pdf]

nature research

Corresponding author(s): Jesuthasan

Last updated by author(s): Jul 8, 2019

Reporting Summary

Nature Research wishes to improve the reproducibility of the work that we publish. This form provides structure for consistency and transparency in reporting. For further information on Nature Research policies, see Authors & Referees and the Editorial Policy Checklist.

Statistics

For all statistical analyses, confirm that the following items are present in the figure legend, table legend, main text, or Methods section.

n/a	Confirmed	
☐	☒	The exact sample size (*n*) for each experimental group/condition, given as a discrete number and unit of measurement
☐	☒	A statement on whether measurements were taken from distinct samples or whether the same sample was measured repeatedly
☒	☐	The statistical test(s) used AND whether they are one- or two-sided<br>*Only common tests should be described solely by name; describe more complex techniques in the Methods section.*
☒	☐	A description of all covariates tested
☒	☐	A description of any assumptions or corrections, such as tests of normality and adjustment for multiple comparisons
☐	☒	A full description of the statistical parameters including central tendency (e.g. means) or other basic estimates (e.g. regression coefficient) AND variation (e.g. standard deviation) or associated estimates of uncertainty (e.g. confidence intervals)
☒	☐	For null hypothesis testing, the test statistic (e.g. *F*, *t*, *r*) with confidence intervals, effect sizes, degrees of freedom and *P* value noted<br>*Give P values as exact values whenever suitable.*
☒	☐	For Bayesian analysis, information on the choice of priors and Markov chain Monte Carlo settings
☒	☐	For hierarchical and complex designs, identification of the appropriate level for tests and full reporting of outcomes
☐	☒	Estimates of effect sizes (e.g. Cohen's *d*, Pearson's *r*), indicating how they were calculated

Our web collection on statistics for biologists contains articles on many of the points above.

Software and code

Policy information about availability of computer code

Data collection	Confocal microscopy was performed using Zen Blue software on a Zeiss LSM800 microscope. Behaviour data was collected using Python codes that are available on GitHub [https://github.com/rkcheng/AlarmSubstance_AdultFish].
Data analysis	Behaviour data was analyzed using scripts written in Python. These are available in GitHub [https://github.com/rkcheng/AlarmSubstance_AdultFish].<br>Imaging data was analyzed using Huygens Professional X11 and Fiji.

For manuscripts utilizing custom algorithms or software that are central to the research but not yet described in published literature, software must be made available to editors/reviewers. We strongly encourage code deposition in a community repository (e.g. GitHub). See the Nature Research guidelines for submitting code & software for further information.

Data

Policy information about availability of data

All manuscripts must include a data availability statement. This statement should provide the following information, where applicable:

- Accession codes, unique identifiers, or web links for publicly available datasets
- A list of figures that have associated raw data
- A description of any restrictions on data availability

Data reported here are available on FigShare [10.6084/m9.figshare.8796695]. This includes raw image files for Fig. 1g and 3s, and tracking data for Fig. 4a-g and 5a-h.

Field-specific reporting

Please select the one below that is the best fit for your research. If you are not sure, read the appropriate sections before making your selection.

☒ Life sciences ☐ Behavioural & social sciences ☐ Ecological, evolutionary & environmental sciences

For a reference copy of the document with all sections, see nature.com/documents/nr-reporting-summary-flat.pdf

Life sciences study design

All studies must disclose on these points even when the disclosure is negative.

Sample size	No sample size calculations were performed for the screen on bacterial lysates. The sample size was chosen based on experience that at least 50% of adult zebrafish normally responded to an active alarm substance.
Data exclusions	Data from the entire set was excluded in behaviour tests where less than 50% of the population responded. This is noted in the Methods section.
Replication	Tests with bacterial and fish lysates that showed a positive result were repeated on a separate set of fish. Pulse chase experiments were replicated on separate fish.
Randomization	Allocation was random
Blinding	Experiments with test substances (bacterial and larval lysate) were performed blinded. The experimenter was unaware of the identity of the substances. Analysis was performed using a computer.

Reporting for specific materials, systems and methods

We require information from authors about some types of materials, experimental systems and methods used in many studies. Here, indicate whether each material, system or method listed is relevant to your study. If you are not sure if a list item applies to your research, read the appropriate section before selecting a response.

Materials & experimental systems

n/a	Involved in the study	
☐	☒	Antibodies
☒	☐	Eukaryotic cell lines
☒	☐	Palaeontology
☐	☒	Animals and other organisms
☒	☐	Human research participants
☒	☐	Clinical data

Methods

n/a	Involved in the study	
☒	☐	ChIP-seq
☒	☐	Flow cytometry
☒	☐	MRI-based neuroimaging

Antibodies

Antibodies used	ΔNp63, cleaved Caspase-3, LC3B, B-catenin,
Validation	ΔNp63 (Santa Cruz SC-8341): Lee, R. T. H., Asharani, P. V. & Carney, T. J. Basal keratinocytes contribute to all strata of the adult zebrafish epidermis. PLoS ONE 9, e84858 (2014).<br>Cleaved Caspase-3 (Cell Signaling Technology 9661): https://www.ncbi.nlm.nih.gov/pmc/articles/PMC3640221/<br>LC3B (Abcam ab48934): Statement on manufacturer website https://www.abcam.com/lc3b-antibody-ab48394.html<br>B-catenin (BD Bioscience 610153): citations given at https://wiki.zfin.org/display/AB/Ab2-ctnnb

Animals and other organisms

Policy information about studies involving animals; ARRIVE guidelines recommended for reporting animal research

Laboratory animals	Danio rerio; Ekwill (for behaviour and alarm substance production), AB (for alarm substance production, pulse chase and antibody label). Aged 3 months - 1 year. Both males and females were used.
Wild animals	N/A
Field-collected samples	N/A
Ethics oversight	IACUC of the Biological Resource Centre at Biopolis, Singapore (#151092) and IACUC of the University of Oregon (#15-98).

Note that full information on the approval of the study protocol must also be provided in the manuscript.
